# Trust and stakeholder perspectives on the implementation of AI tools in clinical radiology

**DOI:** 10.1007/s00330-023-09967-5

**Published:** 2023-07-28

**Authors:** Magnus Bergquist, Bertil Rolandsson, Emilia Gryska, Mats Laesser, Nickoleta Hoefling, Rolf Heckemann, Justin F. Schneiderman, Isabella M. Björkman-Burtscher

**Affiliations:** 1 School of Information Technology, Halmstad University, Halmstad, Sweden; 2 Department of Sociology and Work Science, University of Gothenburg, Gothenburg, Sweden; 3 Department of Sociology, Lund University, Lund, Sweden; 4 Department of Radiology, Institute of Clinical Sciences, Sahlgrenska Academy, University of Gothenburg, Gothenburg, Sweden; 5 Department of Radiology, Sahlgrenska University Hospital, Region Västra Götaland, Gothenburg, Sweden; 6 Department of Medical Radiation Sciences, Institute of Clinical Sciences, Sahlgrenska Academy, University of Gothenburg, Gothenburg, Sweden; 7 Department of Clinical Neuroscience, Institute of Neuroscience and Physiology, Sahlgrenska Academy, University of Gothenburg, Gothenburg, Sweden

**Keywords:** Trust, Artificial intelligence, Clinical decision support systems, Organizations, Radiology

## Abstract

**Objectives:**

To define requirements that condition trust in artificial intelligence (AI) as clinical decision support in radiology from the perspective of various stakeholders and to explore ways to fulfil these requirements.

**Methods:**

Semi-structured interviews were conducted with twenty-five respondents—nineteen directly involved in the development, implementation, or use of AI applications in radiology and six working with AI in other areas of healthcare. We designed the questions to explore three themes: development and use of AI, professional decision-making, and management and organizational procedures connected to AI. The transcribed interviews were analysed in an iterative coding process from open coding to theoretically informed thematic coding.

**Results:**

We identified four aspects of trust that relate to reliability, transparency, quality verification, and inter-organizational compatibility. These aspects fall under the categories of substantial and procedural requirements.

**Conclusions:**

Development of appropriate levels of trust in AI in healthcare is complex and encompasses multiple dimensions of requirements. Various stakeholders will have to be involved in developing AI solutions for healthcare and radiology to fulfil these requirements.

**Clinical relevance statement:**

For AI to achieve advances in radiology, it must be given the opportunity to support, rather than replace, human expertise. Support requires trust. Identification of aspects and conditions for trust allows developing AI implementation strategies that facilitate advancing the field.

**Key Points:**

*• Dimensions of procedural and substantial demands that need to be fulfilled to foster appropriate levels of trust in AI in healthcare are conditioned on aspects related to reliability, transparency, quality verification, and inter-organizational compatibility.*

*•Creating the conditions for trust to emerge requires the involvement of various stakeholders, who will have to compensate the problem’s inherent complexity by finding and promoting well-defined solutions.*

**Supplementary Information:**

The online version contains supplementary material available at 10.1007/s00330-023-09967-5.

## Introduction

Using computer-based decision support systems in healthcare raises several issues, many of which fit the category of trust and trustworthiness. Specifically, it is imperative for professional stakeholders to develop trust in the efficacy of a system for its implementation to succeed [1]. Classical AI models pose a particular challenge in this regard. Their non-deterministic and correlational—rather than causal—nature results in the “black box” problem: the user has no means of scrutinizing the system’s decision process [2, 3]. A scoping review on the future of AI in radiology concluded that a majority of stakeholders disagree with the technocratic prospect of AI replacing human radiologists, and it identified trust as one of the seven determiners of success of AI in radiology [4]. Despite trust being a core requirement for AI in healthcare, little scientific work addresses trust. Gille et al [5] found no consensus on what trust is and how to achieve it in healthcare.

 Few studies address the broad issue of trust in AI for medical imaging [6–9]. They focus mainly on the explainability and interpretability of algorithms as a requirement of trustworthy AI [6, 8, 9]. The common demand for AI models is to be explainable and interpretable so that human experts can understand the reasons for the model output [10]. The previous studies, while providing technical grounds for improving the trustworthiness of an algorithm, do not encompass medical reasoning in the explanations [11].

A broader look at trust and trustworthiness in relation to AI in medical image analysis support could provide grounds for healthcare professionals and other stakeholders to develop appropriate levels of trust towards AI. Hasani et al [7] proposed comprehensive requirements for developing trustworthy AI systems, including stakeholder engagement. They did not, however, cite empirical work in support of this requirement.

Trust depends on the interaction between the involved parties and should be understood as an ongoing process of establishing faith to reduce complexity [12, 13]. The social context is important for the interplay between a trustor and a trustee and consists of activities and strategies that will increase confidence between the involved parties [14]. Human actors come to trust each other or an AI system because of the role a trustee plays in the larger system, such as the organization [15]. Examples of interactive activities to establish propensity to trust [16] are (1) signalling of ability, (2) the demonstration of benevolence, (3) the demarcation of integrity, and (4) the establishment of an emotional connection [17]. Even though emotional connections are important for trust on an interpersonal level, AI in itself should not need to be trustworthy on an emotional level but reflect reliability on the same level as other technologies supporting medical decisions [18].

Taking a starting point in the scarceness of implementations of AI solutions for automatic segmentation of brain lesions on magnetic resonance images in clinical routine [19–23], the purpose of our study was to explore the knowledge gaps surrounding the broad themes of trust in AI, the perspective of stakeholders in AI, and how we can achieve trust in AI in healthcare. We designed an interview study covering a broad variety of stakeholders at one of Europe’s largest university hospitals and collaborating entities. We further aimed to define prerequisites for trust and to identify potential obstacles to achieving trust in healthcare when focusing on AI.

## Materials and methods

**Fig. 1:**
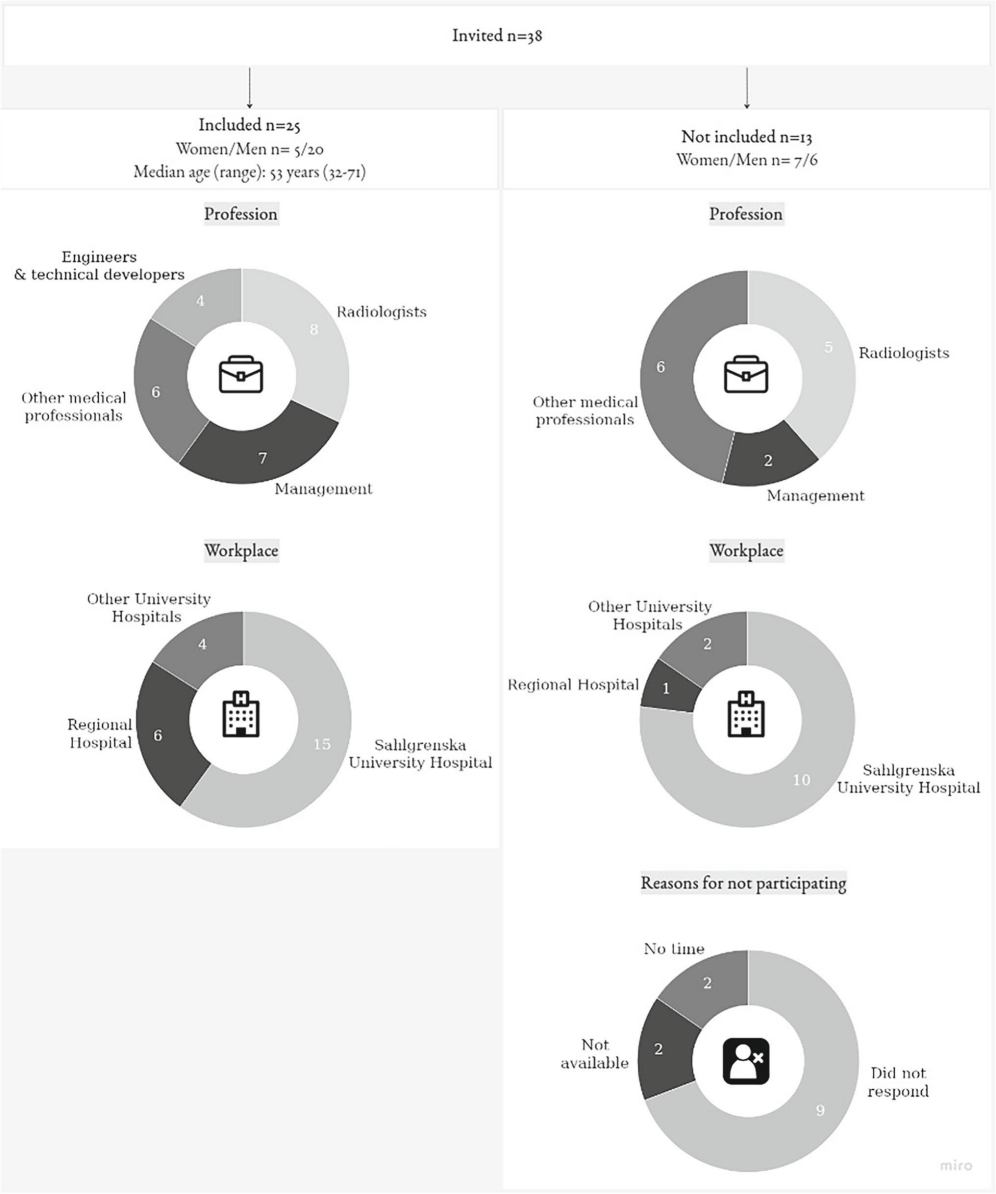
A chart representing demographic information of individuals participating in the interviews of this study and those invited but not participating

Since AI in radiology can be considered a non-typical case [24], a purposive sampling strategy was used. We chose an explorative approach with a focus on how the ongoing development of AI opens for future changes [25]. We aimed to include a variety of respondents with a view to clinical and academic background (medical, technical, and administrative), workplace size, and geographical location. A chart representing demographic information on the 25 individuals participating in the interviews of this study and those invited but not participating (*n* = 13) is given in Fig. 1. The 25 participants held diverse roles in the healthcare system and most medical professionals also held (or had previously held) management or leadership functions and had academic backgrounds. Nineteen of the respondents were directly involved in the development, implementation, or use of AI applications as part of their professional practice related to radiology. Out of 38 invited respondents, 13 did not participate; two radiologists declined the invitation due to lack of time and one radiologist due to leave of absence. In addition, two radiologists, three neurosurgeons, two oncologists, one manager, and one MR-nurse did not respond to the invitation. One manager had resigned from work.

### Data collection

The semi-structured interviews followed an interview guide (Supplement 1) with predefined questions allowing for the possibility to explore unanticipated issues that arose in connection with the data collection [26]. The interviews focused mainly on three themes—development and use of AI, professional decision-making, and management and organization—and included probing questions based on the participant’s responses [27].

The first interview topic consisted of questions on how different types of AI are used or are expected to be used by radiologists. We guided the respondents to stay close to their personal experiences and activities while mapping their use or expectations of AI and challenges related to the specific use of this technology.

The second topic addressed decision-making concerning responsibilities that condition the professional role. The questions pertained to AI and automation of state-of-the-art knowledge, standards, and skills central to the ability to address demands for accuracy, e.g., expert judgment on clinical matters and normative content. The interviews also addressed healthcare professionals’ responsibilities to comply with ethics, standards, and codes regulating their practice as recognized experts [28]. This part of the interview included questions about ambiguities related to accountability and public expectations on clinical reasoning, diagnostic work, and prioritization aligned with broader societal values or perceived common goods.

The third topic of the guide included questions about management and organizational procedures that condition the introduction of automated decision-making (ADM) into professional practice. We focused on the organizational goals and evaluations of administrative efficiency, fairness, quality, and safety issues linked with ADM. These questions were of interest in relation to many previous studies showing how managerial issues lead to the marginalization of professionals’ ability to make informed judgments [29]. By asking managers how they frame ADM, we intended to identify how organizational conditions shaped the ability to translate knowledge, codes, and standards to the needs and features of the case at hand [28]. We were thus able to identify further ambiguities conditioning professional discretionary capabilities.

The interviews were performed by two social scientists—not earlier working with specific neuroradiology-related questions to decrease interpretation bias (M.B. and B.R.)—recorded, and transcribed by an external transcriptionist. A logbook was kept in connection with each interview to record the investigators’ initial impression of the data.

### Data analysis

**Fig. 2:**
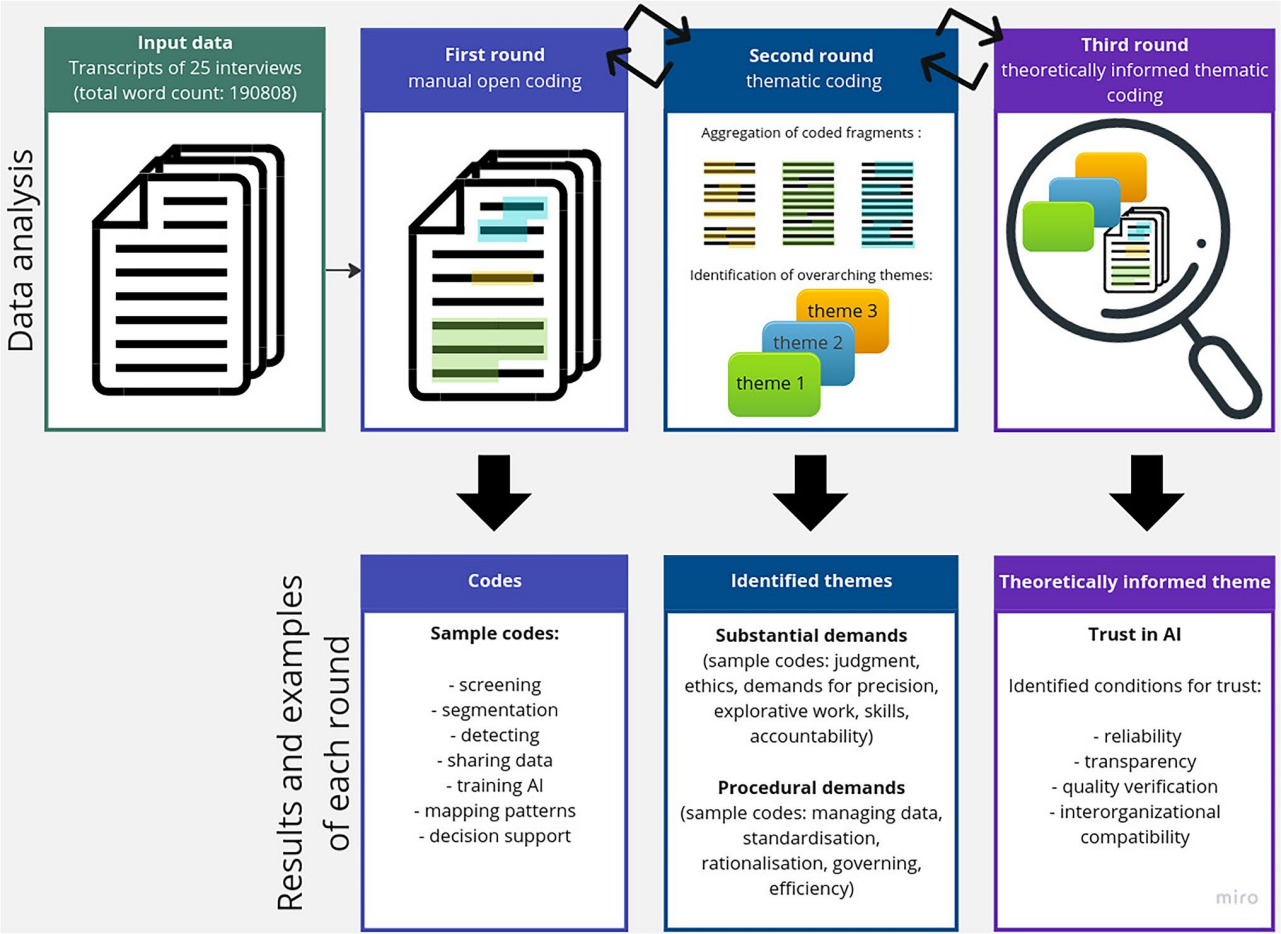
Coding procedure leading to the identification of conditions for trust in AI. In the first round, open coding was performed by tagging of words/text fragments in the input transcript data with concrete empirical and in vivo codes. In the second round of analysis (thematic coding), we aggregated existing codes to identify how the range of activities involving AI was linked with broader clusters of meaning related to professional and organizational norms, values, rules, and policies. During this round of analysis, we identified themes linked with substantial dimensions of clinical work and procedural challenges. In the third round (theoretically informed thematic coding), we iteratively re-read codes, text fragments identified in the previous rounds, and the transcripts. Thereby, we established theoretically informed themes leading to identification of conditions for trust in AI. The examples of raw data quotes, and more information on the identified theoretically informed themes and related conditions for trust in AI are given in Tables 1, 2, 3, and 4

We used the ATLAS.ti Web, Scientific Software Development GmbH (https://atlasti.com/) (AI add-ons recently available for the software were not used in this study) to identify, retrieve, and reflect on statements in the transcripts, applying and clustering codes in an iterative three-phase coding procedure (Fig. 2). In the first coding round, we kept close to the interviewees’ actual statements using concrete empirical and in vivo codes. In the second round of coding, we aggregated existing codes to identify how the range of activities involving AI was linked with broader clusters of meaning related to professional and organizational norms, values, rules, and policies. During this round of analysis, we identified themes linked with substantial dimensions of clinical work and procedural challenges. The third round of coding involved a re-reading of codes and themes based on theoretical reflection.

## Results

Of 912 coded text segments, 265 were directly related to aspects of trust. The iterative three-phase coding process is illustrated in Fig. 2. During open coding, concrete empirical and in vivo codes were defined e.g., visualizing, screening, segmentation, detecting, teleworking, free text, data sharing, managing data, training AI, mapping patterns, and decision support. The second coding round—thematic coding—resulted in identified themes linked with substantial dimensions of clinical work, e.g., judgment, ethics, demands for precision, exploration, skills, and accountability, and themes linked with procedural challenges, e.g., importance of standardisation, rationalisation, governance, and efficiency. In the second thematic coding round of the analysis, we identified trust as a recurring theme that emerged both on a local level in the radiologists’ practice and on a central organizational level connected to managerial and organizational demands.

The analysis of the interviews resulted in four theoretically informed themes of trust: *trust in relation to reliability* (64 codes); *trust in relation to transparency* (61 codes); *trust in relation to quality verification* (59 codes); *trust in relation to inter-organizational compatibility* (81 codes). The themes fit in two dimensions of trust: i.e., trust in substantial requirements and trust in procedural requirements. Substantial trust relates to trust in data, methods, infrastructure, and the like. Procedural trust relates to requirements that raise technical, organizational, and administrative challenges.

In Tables 1, 2, 3, and 4, we present and define the conditions under which the constituent aspects of four themes of trust generate trust in practice for our interviewees. The four themes are trust in relation to reliability, trust in relation to transparency, trust in relation to quality verification, and trust in relation to inter-organizational compatibility. Each aspect is supported by a quote from the interviews as an example of our definition of trust in practice.

**Table 1:** Aspects and conditions for trust in relation to reliability (substantial requirement)

Aspect	Condition for trust	Example quotes (translated from native language)
Volume	AI systematically provides a dependable basis for diagnostics in large datasets.	“The more information we have, the more we need to screen, and the risk increases that we miss something, especially if it is not directly asked for in the referral, but becomes visible in the image, such as the small tumour that is not directly related to trauma.”
Granularity	AI accurately and efficiently extracts clinically relevant information from highly detailed information.	“Screening is about analysing very large volumes of individuals. And therefore, we felt that this is not feasible in Sweden or anywhere else […]; And we can diagnose smaller tumours than we could see before [with AI]. We can identify incidental findings when we do these examinations that we didn’t know existed, but they are visible with these new methods.”
Bias	AI increases radiologists’ awareness of possible cognitive bias in decision-making.	“Technology can obviously become a risk in those cases, i.e., when it generates too much information to manage. Still, it may support your judgment as a doctor, i.e., helping you to become a better doctor, considering the relevant test results, or simply what we need to do.”

**Table 2:** Aspects and conditions for trust in relation to transparency (procedural requirement)

Aspect	Condition for trust	Example quote (translated from native language)
Standards	Evidence-based support that minimizes bias due to differences in competence and degree of experience of the clinicians.	“It should not matter whether an image is diagnosed in Linköping or at Sahlgrenska. A doctor with less experience should have support from a machine that says, “this looks like a normal brain”, or “this looks like a tumour”. It should always use the same methods.”
Traceability	The rules by which AI works can be followed by the domain professionals.	“A radiologist should be able to know that “yes, this algorithm followed these rules, and I trust that it actually followed the rules, so I can click okay.” AI can identify complex relationships in the data that are difficult for a human to understand. So, that’s a challenge: how can these complex relationships be presented so that the human can see that it is evidence-based, which is required in the medical profession? That is essential if we should be able to trust the automation systems that we build.”
Explainability	The informed interaction of humans and (explainable) medical AI enhances the diagnostic ability and accountability of radiologists.	“As a radiologist I have a list with examinations. Then I would like to have a system that says, ‘look at these first, because here is something’, and then automatically sorts out the ones that it defines as ‘normal’.”

**Table 3:** Aspects and conditions for trust in relation to quality verification (substantial requirement)

Aspect	Condition for trust	Example quote (translated from native language)
Methodological rigour	Ensuring a certain level of quality by reducing human variation through providing unbiased, evidence-based diagnoses that can be evaluated.	“We showed that this AI algorithm was as good as the average radiologist when left to work on its own. It was never better than the best radiologist, but better than 40–60% of the radiologists.” “Segmentation must be carefully done. It takes a qualified nurse several hours to correctly draw the organs. We have methods but there are human variations. The nurse must correct errors. [An AI] does it approximately exactly the same way each time, with less variation than a human. Patient safety is ensured or even increased. If we can get a more similar work practice between hospitals in Sweden and globally, that would benefit the patient and healthcare.”
Local validation	Implementation sensitive to variations in local modalities’ work processes, and demographic characteristics developed through human-machine learning.	“We may need one AI system for one type of variable, such as a finding, and another AI system for another variable, such as age group. We may need several different AIs to identify different types of illness in the same organ. That generates a tricky situation. All AI systems must be validated and tested clinically in our environment. We cannot just buy them and trust them.” “We really need to test the algorithms and use a representative dataset. We need a relevant dataset to be able to evaluate the algorithm.” “I get biased if the AI already suggested that the sample is normal. Then I need to make an even greater effort to overcome bias. The radiologist must train jointly with the AI and we must study how that works. I’d say that is the most important reason why we have not succeeded in implementing [the technology] yet. We don’t know how radiologists work in combination with AI.”

**Table 4:** Aspects and conditions for trust in relation to inter-organizational compatibility (procedural requirement)

Aspect	Condition for trust	Example quotes (translated from native language)
Capacity	Data from different sources shared and integrated into a coherent infrastructure that leverages the organization’s capacity to plan, distribute, follow-up, and evaluate on an organizational level.	“… we need good integration … between the source systems, or the modalities, if we are talking about images. Sometimes … we want to access … existing produced data, via delivery from journal systems. And sometimes we produce our own data, in the studies. If it’s lab data or pictures or something. And then there is of course … regulated processes then, where you may have to leave the Patient Data Act and switch to GDPR instead.” “… it’s a scale, on the one hand it’s almost, you could say a safe area to work with AI solutions, when it comes to production flows, logistics and material flows and stuff like that. And economics, human relations. There are lots you could cover…and then you have what many of the others who really work with diagnostics and treatment are doing and … there are completely different conditions. And we should try to be some kind of generalist specialists, knowing very much, or very little about very, very, very much. With an in-depth knowledge of, for example, ethical review, data sharing, patient safety and medical evidence and such. We cannot do that, without people like the specialists and others who have to help, like lawyers. We certainly have lawyers with us too, but … but, yes, it’s very exciting.”
Control	Organization has control over data.	“… you can read to some extent how many get this type of treatment, and you can see regional differences for example. In a way that can then increase the chance, at least for equal care. So, it’s more the kind of question whether … do we provide surgeries to a lesser extent or not, and so on. What does it depend on? And then, you can go in and watch the data. This is because there is more of that type of cancer here. Yes, yes but then it explains why. Or no, here we should change the procedure and you should follow the care program better. Maybe identify that the activities are governed by too many local traditions, and so on.”

## Discussion

We identified four themes related to trust that are classified as substantial or procedural requirements. Developing solutions to the requirements demands participation from all stakeholders, in particular professionals using the technology. We further need to foster an organizational awareness of the importance of trust and collaboration of developers, users, regulators, and managers [5, 12]. As clinical implementation of AI in radiology is in its infancy, we must address concerns about developing appropriate levels of trust in AI to allow well-balanced clinical decisions based on automatically generated information [30, 31]. Developing such trust forces radiologists and other healthcare specialists to reflect on the consequences of including AI in professional judgment and decision-making in clinical practice, for instance, when AI solutions use combinations of retrospective and real-time health data to support evidence-based decision-making, individualized care, and precision medicine [32–34].

The reliability of AI is crucial to trust. We identified three aspects of reliability: volume, granularity, and bias. When examining large *volumes* of data, AI is expected to provide a dependable basis for diagnostics [35]. Access to increasing amounts of image data can create better diagnoses, but there is also a risk of information overflow. Reliability is generated when AI systematically returns predictable output in a large dataset. *Granularity* refers to how increased depth of information could result in higher precision in detecting findings given available resources to process and analyse the data. For example, the technological advances in imaging modalities lead to increased resolution or new types of available diagnostic images [36]. Those technological improvements can benefit patients only if the detailed information can be processed and analysed promptly. AI’s ability to accurately extract clinically relevant information from highly detailed information increases its reliability. The third identified *reliability* aspect is *bias*, i.e., the risk of being misled by preconceptions. AI’s ability to compare the current case with all existing reference cases increased the radiologist’s awareness of possible cognitive bias in decision-making [37]. By providing a second opinion, AI made the radiologist aware of potential bias. An example given by a radiologist in the interviews was that the more recent cases tended to influence them the most, whereas the AI considered all cases it had been trained on and thus provided them with a more extensive frame of reference [38].

Trust based on transparency draws on the radiologists’ understanding of the AI’s “inner workings” when handling individual cases [38]. We identified three themes crucial to transparency: standards, traceability, and explainability. *Standards* refer to how the AI can connect different cases to enhance the radiologists’ understanding of how data is managed so that the output becomes transferable to new cases. Standards make it possible to transfer insights from one case to another by providing evidence-based support that minimizes bias due to differences in competence and degree of experience. When AI becomes a trusted standard, we expect that the quality of diagnostics will improve in general. *Traceability* was an inherent aspect of standards as an interviewed radiologist argued that to be able to trust how the algorithm is processing data, the basis for making a decision must be traceable by domain professionals [39]. The requirements for standards and traceability lead to the third identified theme related to transparency: *explainability*. Explainability, defined as the ability of an AI system to provide a clear and understandable explanation of how it reached a particular decision or conclusion [40], enhances the increased diagnostic ability of radiologists as an informed interaction of humans and medical AI [41].

Various AI applications may require various degrees of trust towards the tool. Both traceability and explainability may be particularly important in scenarios, where the prediction of AI cannot be easily verified. For example, when AI is used for segmentation, physicians likely do not need the same degree of trust towards the tool since the outcome can be visually assessed. However, if for example dataset or distribution shift is present, it may not be feasible or even possible for the individual physician to verify the accuracy of the outcome to the same extent. Instead, physicians must develop appropriate levels of trust towards the support system. Therefore, other validation strategies based on traceability and explainability of the system are necessary to develop appropriate levels of trust towards AI.

Organizational procedures for quality verification in diagnostic work foster trust-based *methodological rigour* and *local validation*. Methodological rigour underpins trust when AI emerges as an organizational means. Trained on accurate data, AI “never gets tired and never makes mistakes”, addressing interviewees’ concerns for variations in diagnosis quality over time [39]. “Verified data sets are crucial to provide valuable support as references or maps guiding the radiologist”. At the same time, the interviewees point out that a challenge of verifying data is that the algorithm learns from standardized datasets and therefore lacks the ability to adapt to local knowledge [42]. The second theme addressing quality verification serving trust in AI was the need for a *local validation* process sensitive to variations in modalities and work processes. Local demography requires datasets specific to that particular region or cohort and cater for differences between modalities, even if they come from the same manufacturer. It was suggested that human-machine learning was needed to deal with a potential bias from the data and how it influences radiological evaluation.

The results show that radiologists’ trust in AI depends on the experience that AI is compatible with other systems and practices in the organization, increasing their capacity and providing control [43]*. Capacity* means that data from different sources is shared and integrated into a coherent infrastructure that leverages the organization’s capacity to plan, distribute, follow-up, and evaluate on an organizational level. Data sharing is crucial both within organizational units, between different hospitals, nationwide, and internationally to gain capacity. Trust in AI emerges when a variegated range of data formats are integrated into existing modalities so that experts across organizational or functional boundaries can share and use data to collaborate efficiently and safely. Integrated data must be coherent to support the management of the healthcare organization. However, in some cases, legal requirements regarding e.g. patient journals, personal data, and professional secrecy complicate control and validation procedures by creating tension between efficiency and patient integrity. To make AI increase trust in capacity building, the organization must have *control* over data. Variegated data sources and work processes make comparisons difficult, potentially delimiting trust. Having control over the data is also essential for monitoring the dataset distribution shift; continuous learning of the AI system on new data may lead to gradual change in the predicted outcomes. The organization must ensure though that this shift does not occur due to the bias in the training data.

To summarize, based on inter-organizational compatibility, trust in AI emerges when standardized procedures to follow-up, manage, and evaluate are fair, legal, and secure.

This study comes with certain limitations that could constrain the generalizability of the findings in a different context. The interviewees were selected purposively, resulting in a selection bias, which limits the results to their perspectives only. Furthermore, we used an explorative approach and open coding to analyse the interviews instead of consolidated criteria. While this approach allows for a freer exploration of the topic, it also comes with a risk of biased answers and misunderstanding of the topic between the interviewers and interviewees.

## Conclusions

Trust in AI in healthcare is a complex attitude that builds on various procedural and substantial demands. To define the requirements that promote trust in AI, trust can be approached as a leap of faith rather than absolute certainty, as the latter may not be achievable or even desirable in this context. The procedural and substantial demands for trust identified in this study are conditioned on aspects related to reliability*,* transparency, quality verification, and inter-organizational compatibility. Each of these aspects is further divided into specific conditions that must be fulfilled. Creating the conditions for trust to emerge requires the involvement of various stakeholders, who will have to compensate the problem’s inherent complexity by finding and promoting well-defined solutions.

### Supplementary Information

Below is the link to the electronic supplementary material.

Supplementary file1 (PDF 132 KB)

## Abbreviations

ADM: Automated decision-making
AI: Artificial intelligence

## Declarations

### Guarantor

The scientific guarantor of this publication is Isabella M. Björkman-Burtscher.

### Statistics and biometry

No complex statistical methods were necessary for this paper.

### Methodology

• exploratory study

• performed at multiple institutions
