# Supplementary material for: Trust and stakeholder perspectives on the implementation of AI tools in clinical radiology
Source: Eur Radiol. 2023 Jul 28;34(1):338–47. doi: 10.1007/s00330-023-09967-5 (PMC10791850; doi:10.1007/s00330-023-09967-5)
Supplement: Supplementary file 1 — Supplementary file1 (PDF 132 KB) [file 330_2023_9967_MOESM1_ESM.pdf]

Trust and stakeholder perspectives on the implementation of AI tools in clinical radiology

Electronic Supplementary Material

Supplement 1 -- Interview guide

This document is a translation of the semi-structured interview guide from the original language to English. The interview guide is not validated and adjustments to research questions, potential cultural aspects and local context might be needed before reusing it. Although the defined questions are listed, the format of a semi-structured interview allows for the possibility to explore unanticipated issues that arise in connection with the data collection. Respondents are guided to stay close to their personal experiences and professional activities while mapping their use or expectations of AI. The term "technology" is used to cover any technique referred to as AI during the interview.

Questions for interviews

1. Characteristics and affordances of the technology
   - 1.1. What type of technology is used/do you use and what type of findings do physicians/do you have to consider when making a decision?
   - 1.2. How does AI generate the findings?
   - 1.3. What type of data constitutes the basis for generating the findings?
   - 1.4. How is this data presented?
   - 1.5. How do you envision that the physician/you will use the technology?
   - 1.6. How does the technology make a difference and in what cases?

2. Developing the technology
   - 2.1. Which actors are involved in the actual development/implementation of the technology?
   - 2.2. How does the communication take place between the involved actors? Are any potential errors in the design dealt with and how?
   - 2.3. How do you explain to the engineer/technician what you need and how you work?

3. Professional judgment/Accuracy (substantial dimension)
    3.1. How does the physician relate to the interface/what features are used?
    3.2. How does the physician consider findings and what challenges arise when a physician has to assess the findings and make a diagnosis/decision?
    3.3. What challenges arise when a physician makes decisions about how the diagnosis/radiological finding should be handled? Are there any additional pieces of information, routines, or regulations that, in addition to the findings, shape the decision?
    3.4. Do any prioritisation issues arise with regard to establishing a diagnosis or making a treatment decision?
    3.5. Do physicians/experts discuss these challenges among themselves in any way?
    3.6. Is any additional information from or communication with the patient involved in any way?
    3.7. Are there any challenges that can be linked to the technical design?

4. Administrative dimension (procedural dimension)
    4.1. How do physicians document and communicate their decision/diagnosis within the organization)?
    4.2. How does the physician communicate with the patient (direct, indirect or mediated)?
    4.3. Does the design of AI enable the possibility to follow-up on mistakes made in diagnosis/decision and how?
    4.4. Do the physicians discuss and handle the risks of possible incorrect decisions/diagnoses already made based on the technology? If so, how?
    4.5. Does the organization follow up on possible incidents?
    4.6. What are the success factors (obstacles) for the technology to make a breakthrough in the organization (stakeholders, regulations, infrastructure, existing working methods)?

5. Supplementary questions for interviews with management or leadership (strategy)
   5.1. In which areas of application can AI technology have the greatest effect?
   5.2. What dangers/challenges do you see with the technology?
   5.3. Does the design of AI enable monitoring and quality control on an organizational level?
   5.4. What does AI in healthcare mean in the longer term (efficiency, quality, standardization, equality)?
   5.5. What are the success factors (obstacles) for the technology to reach a breakthrough in the organization (stakeholders, regulations, infrastructure, existing working methods)?
